# Families' View on Primary Nursing in Intensive Care Units ‐ A Cross‐Sectional Study

**DOI:** 10.1111/nicc.70575

**Published:** 2026-07-14

**Authors:** Lars Krüger, Francesco Squiccimarro, Thomas Mannebach, Almut Pörner, Benjamin Sarx, Christian Siegling, Esther Mertins, Tobias Becker, René Schramm, Jan Gummert, Volker Rudolph, Laura‐Carina Kurz, Christian Höke, Franziska Wefer, Gero Langer

**Affiliations:** ^1^ Ruhr University Bochum, Heart and Diabetes Center NRW, Care Directorate, Project and Knowledge Management/Care Development Intensive Care Bad Oeynhausen Germany; ^2^ Institute of Health, Midwifery and Nursing Sciences, Medical Faculty Martin Luther University Halle‐Wittenberg Halle (Saale) Germany; ^3^ Ruhr University Bochum, Heart and Diabetes Center NRW, Surgical Intensive Care Unit Bad Oeynhausen Germany; ^4^ Ruhr University Bochum, Heart and Diabetes Center NRW, Care Directorate, Care Development Bad Oeynhausen Germany; ^5^ Ruhr University Bochum, Heart and Diabetes Center NRW, Care Directorate Bad Oeynhausen Germany; ^6^ Ruhr University Bochum, Heart and Diabetes Center NRW, Department of Thoracic and Cardiovascular Surgery Bad Oeynhausen Germany; ^7^ Ruhr University Bochum, Heart and Diabetes Center NRW, Department of General and Interventional Cardiology/Angiology Bad Oeynhausen Germany; ^8^ Ruhr University Bochum, Heart and Diabetes Center NRW, Cardiology/Angiology Intensive Care Unit Bad Oeynhausen Germany

**Keywords:** family nursing, nursing, nursing process, patient‐centred care

## Abstract

**Background:**

Family‐centred care (FCC) has come into the focus of treatment in intensive care units (ICUs). Primary nursing (PN) as a patient‐centred care nursing organisation model seems to be a good prerequisite for the integration of patients' families into nursing care. Current research on families' perspectives of PN on ICUs is rare.

**Aims:**

To evaluate satisfaction of families with PN, compared with individual nursing as standard care (SC). Secondary aims were to evaluate nursing framework conditions, support by physicians, interprofessional collaboration and environmental factors of the hospital.

**Methods:**

Cross sectional study with patients' family members in a surgical and medical ICU. A validated questionnaire with 27 items and 3 or 5‐point Likert scales was used. The Wilcoxon rank test was used to calculate group differences. Data collection took place between November 2023 and May 2025.

**Results:**

Overall, 213 questionnaires were incorporated into the analysis. Family members of patients in PN (*n* = 63) reported better information about care measures, with a median of 1 (IQR 1 to 2) on a 5‐point Likert scale (1 = very good; 5 = very bad) compared to 2 (IQR: 1 to 3) in SC (*n* = 150; *p* = 0.047). They also reported better information to help support patients' recovery (PN: 1 [IQR: 1 to 2]; SC: 2 [IQR: 1 to 3]; *p* = 0.01). Nursing framework conditions were similar in both groups, as well as most of physicians' support of families, the collaboration of interprofessional staff on ICU and environmental factors of the hospital.

**Conclusions:**

Families within our study had contact to PN or SC. Positive results in both groups are in line with how PN and also individual nursing are described in the literature. Follow‐up studies using a mixed‐methods design are necessary to further investigate the effects of PN.

**Relevance to Clinical Practice:**

Family members ranked PN and SC positively, with trends for PN. This could indicate that FCC is being carried out on both ICUs.

## Impact Statements

- What is known about this topic?
  ○ As a patient‐centred nursing organisation model, primary nursing (PN) includes the patients' family in the nursing care process.
  ○ There are published research projects on PN that provide first indications of positive associations in families on ICU.
- What this paper adds
  ○ Families' views of patients cared for in PN with continuous nursing care by a primary nurse from admission through discharge or individual nursing as standard care without continuous nursing care on two intensive care units (ICUs) were studied.
  ○ Compared to individual nursing as standard care on ICU, families in PN were better informed about care measures and were better able to support patients' recoveries.
  ○ Collaboration of the interprofessional staff on ICU was ranked positively by families in both PN and individual nursing as standard care.

## 1 Introduction

Family‐centred care (FCC) has increasingly become part of multi‐professional treatment on intensive care units (ICUs) in recent years [1, 2]. As a nursing organisation model, primary nursing (PN) focuses on person‐centred care [3] and also includes the families of patients.

## 2 Background

In 2007, the Society of Critical Care Medicine (SCCM) published a first guideline for FCC, which was revised in 2017 [4] and 2025 [1]. According to the guideline, the term family should be defined by the patient, and family members do not have to be related to him or her [4]. FCC emphasises the needs and values of individual families and family members as central to care delivery [4].

The outbreak of the COVID‐19 pandemic also had a negative impact on interaction with the patients' family on ICUs [1, 5]. Especially at the beginning of the pandemic, some recommendations for FCC, like open visiting policies [1, 4], were not practiced [5]. The integration of families in nursing care, for example, with the help of adapted and understandable communication, receiving information strategies, open visiting policies and involvement in patient care, are associated with a better satisfaction of families [6, 7]. Moreover, among other outcomes, a decreased level of family stress [6, 8] and reduced patient delirium [9] were reported. For this reason, support for families is an important aspect of interprofessional care on ICU [10] and especially for nursing [8, 11]. Certification programmes for family‐friendly ICUs have already been described for this purpose [12].

On ICUs, different nursing organisation models are practiced [13, 14, 15]. Individual nursing as one model provides patient‐centred care [16]. One nurse is responsible for the nursing care of assigned patients during one shift, while the overall responsibility for the nursing care process remains with the nursing leader [16]. The nursing organisation model PN is described as the most patient‐centred form of nursing organisation [3, 16]. In PN, the primary nurse is responsible for the overall nursing care process and an associated nurse follows the written nursing care plan during periods of absence. PN consists of core elements: responsibility for relationships and decision‐making, work allocation and assignment of patients, communication between employees and management, and leadership philosophy [17]. The areas of relationship building and communication in particular also affect families of patients on ICU [18, 19, 20, 21]. In PN the primary nurse is responsible for the nursing care process—ideally from admission to discharge [16] from the ICU. This also includes a comprehensive structured assessment of the nursing needs and social history of the patients. PN is internationally practiced on ICUs [15, 18, 22, 23]. Emerging research indicates that the implementation of PN on ICU may enhance the better integration of families into nursing care [18, 24, 25]. Four years after implementation of PN, Manley et al. [25] identified improved satisfaction among patients' families with nursing care, based on focus group interviews (FG) with nurses. Three years after implementation of PN, Goode and Rowe [18] also conducted FG with nurses and indicated that the primary nurse, as a trusted caregiver, may help reduce anxiety and restlessness of family members. Krüger et al. [24] interviewed nurses in focus groups before and during the implementation process of PN on ICU. Nurses described family members as an important resource in nursing care and reported that PN actively involved them in the nursing care process [24].

Studies that focus on PN from the perspective of families on ICU are rare. Only one randomised feasibility study could be found in which a small group of family members were surveyed using a questionnaire after the implementation of PN as a secondary outcome [26]. As a result, among others, it reported a better influence on the patient's care [26].

### 2.1 Aims

Primary aim of this study was to evaluate satisfaction of families with PN, compared with standard care (SC). Secondary aims were to evaluate nursing framework conditions, support by physicians, interprofessional collaboration and environmental factors of the hospital.

## 3 Methods

**FIGURE 1:**
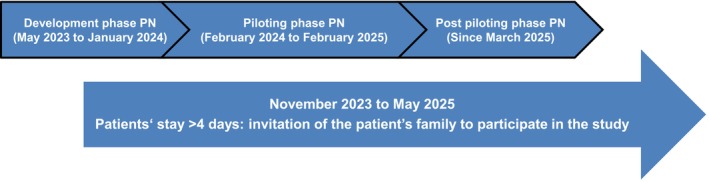
Overview of the evaluation of piloting primary nursing. Abbreviation: PN, Primary Nursing.

The report of this cross sectional study follows the Strengthening the Reporting of Observational Studies in Epidemiology (STROBE) [27] (Table S1). PN as a nursing organisation model with roles of primary nurses and associated nurses was developed in line with the Complex Intervention Framework of the Medical Research Council guidance (MRC‐Framework) [28]. Results of the implementation process were published elsewhere [22]. This study presents a further evaluation with a focus on patients' families in the context of PN (Figure 1).

### 3.1 Setting

The study was conducted on two ICUs at the Heart and Diabetes Center NRW, a German university hospital of the Ruhr University Bochum. ICU 1 has 25 beds with a focus on patients after thoracic and cardiovascular surgery. ICU 2 has 23 beds and focuses on patients with general and interventional cardiology or angiology treatment. In both ICUs, PN had been practiced since February 2024. The nurse‐to‐patient ratio is 1:2. Nurses in both ICUs are registered nurses with a basic training of 3 years, a Bachelor's or Master's degree in nursing or further training like a German state‐certified further training course in intensive and anaesthesia care nursing (ICU training), nursing education, respiratory therapy or palliative care. Both ICUs have regulated visiting hours in the afternoon (2–6 PM), while additional visits may be arranged after personal consultations with nurses and physicians. ICU 1 also practices weekly nursing visits. On ICU 2, nursing visits were implemented in 2026. Family members are currently not included in nursing visits. In accordance with the recommendations for FCC [1], both ICUs also offered an informational flyer about the patients' ICU stay, a waiting area for family members, and access to spiritual and/or psychological support.

### 3.2 Participants

We invited a convenience sample of patients' families over the time period of 19 months (Figure 1). Participants were eligible if they were (i) at least 18 years old, (ii) had visited the patients on‐site on ICU, (iii) were family members of patients with an ICU stay exceeding 4 days, (iv) had sufficient German language proficiency and (v) gave their written informed consent. Only one family member per patient could be included. Participants were excluded if they had not received personal study information from the study team on‐site.

### 3.3 Primary Nursing

PN is implemented for patients with an ICU stay of at least 3 days. Primary nurses and associated nurses have specific tasks which were developed in several working groups before implementation and published in detail elsewhere [22]. The four core elements of PN serve as the foundation for nurses' practice and decision‐making. Primary nurses must be registered nurses with a minimum of 3 years of professional experience on ICU. Ideally, nurses also have formal ICU training or a Bachelor's degree [22]. In cases of part‐time employment (20–25 h per week), nursing leaders assign a primary nursing tandem to ensure continuous and consistent patient care.

The responsibilities of primary nurses include, among other things, the nursing care process in theory and practice with max. two patients [22]. To fulfil this task, a comprehensive structured assessment of the nursing needs and social history of the patients as the first step in the nursing care process is necessary to generate a written nursing care plan [29]. Moreover, primary nurses are in close communication with both the interdisciplinary team and the patient's family. Within the PN model, patients and their families are informed about the responsible primary nurse during a bedside conversation and an additional flyer. If family members are not present on a daily basis, they will be notified by phone and an in‐person appointment will be scheduled. If the primary nurse is not available, for example, in case of vacation or a different shift, the associated nurse assumes the nursing care plan [22].

### 3.4 Standard Care

Individual nursing as SC was practiced in both ICUs before PN started. In case of an ICU stay less than 3 days or a lack of nurses in the role as primary nurses, standard care is still practiced. In SC, no written nursing care plan is available, and the structured assessment of nursing needs and patients' social history is applied only partially and conveyed orally. Moreover, there is a change in the responsible nurses in every shift.

### 3.5 Evaluation and Sample

We used a shortened version of a validated German questionnaire by Huber et al. [30] in a pen‐and‐paper format. The questionnaire was developed for family members of geriatric patients in Switzerland to research how satisfied they are with hospital care and nursing care. The questionnaire was also used in ICU settings before [26]. Internal consistency reliability was calculated for different areas (nurses, physicians, environmental factors) of the questionnaire. Cronbach's alpha for overall areas of the questionnaire was 0.71–0.92 [30].

The used questionnaire included 27 items and a concluding free text field at the end, presented across three pages. There are three items for baseline characteristics. Altogether, 16 items address satisfaction with nursing care and nursing framework conditions, including, for example, explanations of how to use the room bell and the availability of a nurse as a permanent contact.

Furthermore, five items evaluate physician support and interprofessional collaboration, and an additional three items focus on environmental factors, such as the hospital reception and the patient room setup. Altogether, 21 items were Likert scales with 3 (*n* = 3) or 5 items (*n* = 18) as an answer option. For example, one question was ‘Did you receive information material about the patient's stay on ICU?’ with the answer options being: 1: yes, 2: partly, 3: no. An example of a question with a five‐point Likert scale was ‘Can you have a say in how your relative's personal care is organised?’ with the answer options 1: yes, completely to 5: no, not at all. For all items with Likert scales, it was also possible to choose ‘I cannot answer that’ when appropriate.

The questionnaire was formatted according to the hospital's design using TeleForm software, enabling automatic scanning after completion.

### 3.6 Recruitment and Data Collection

The study took place from November 2023 to May 2025. Overall, 213 questionnaires could be analysed. A research team of four nurses and an additional local study leader were responsible for the information on the study, informed consent of participants, and data collection on both ICUs. All nurses were trained for informed consent and data collection by the first author and overall study leader (LK). One nurse in each study group had a Bachelor's degree in nursing. Local study leaders still studied in a Bachelor's degree programme in nursing science (BS, CH).

Data collection started 3 months before the implementation of PN (Figure 1). Participants were provided with verbal and written information and were personally approached by a member of the research team during visiting hours. Participants gave their written informed consent before data collection. The questionnaire was completed by the participants in the absence of the research team on site and then placed in a sealed box in both ICUs or returned by mail. Members of the research team were available to address any questions.

### 3.7 Statistical Methods

The software TeleForm was used for scanning the questionnaires by TB and LK. The system was configured with high sensitivity, allowing TeleForm to flag imprecisely marked responses, which then required manual validation (LK). In cases of ambiguity, a second researcher (TB) was consulted. At least 50% of each item had to be answered to be evaluable for this study. Questionnaires completed by family members prior to the implementation of PN were assigned to the SC group during data analysis, as the study time period was too short to be evaluated as part of a pre–post design.

We summarised categorical variables as percentages and number of observations, and continuous variables as median with interquartile ranges (IQR). Questions that participants answered with ‘I cannot answer that’ were listed separately as unanswerable and were reported separately. All variables were non‐normally distributed, as indicated by the Shapiro–Wilk test. If possible, we used the Wilcoxon rank test to calculate group differences in ordinal variables (five or three‐point Likert scale). *p*‐values < 0.05 were considered statistically significant. All analyses were performed using R Statistical Software (v4.3.3; R Core Team 2025).

### 3.8 Ethical Considerations

Written informed consent was given by all study participants. The ethics committee of the Medical Faculty of the Ruhr University Bochum (2022–987), based in East Westphalia, approved the study on 18 November 2022.

The study was subsequently registered in the German register of clinical trials as (DRKS‐ID: 00030966). All described investigations involving human beings were carried out in accordance with national law and the Declaration of Helsinki from 1975 (in the current, revised version).

## 4 Results

 Altogether, 24 items were sufficiently completed (> 50%) and could be included. In 29.58% (*n* = 63) of cases, PN was practiced, and in 70.42% (*n* = 150) SC, respectively. Most participants were the spouses of the ICU patients in PN (63.49%, *n* = 40) and SC (52.67%, *n* = 79). The frequency of ICU visits was in median ≥ 2 times/week in both groups, and the patients were still on ICU while the questionnaire was being completed (Table S2). In PN, patients stayed in median 26 days (IQR: 15 to 42 days) on ICU, compared to 12 days (IQR: 7 to 24 days) in SC and they received a more intensive ICU therapy (Table S3).

### 4.1 Satisfaction of Families and Nursing Framework Conditions

**FIGURE 2:**
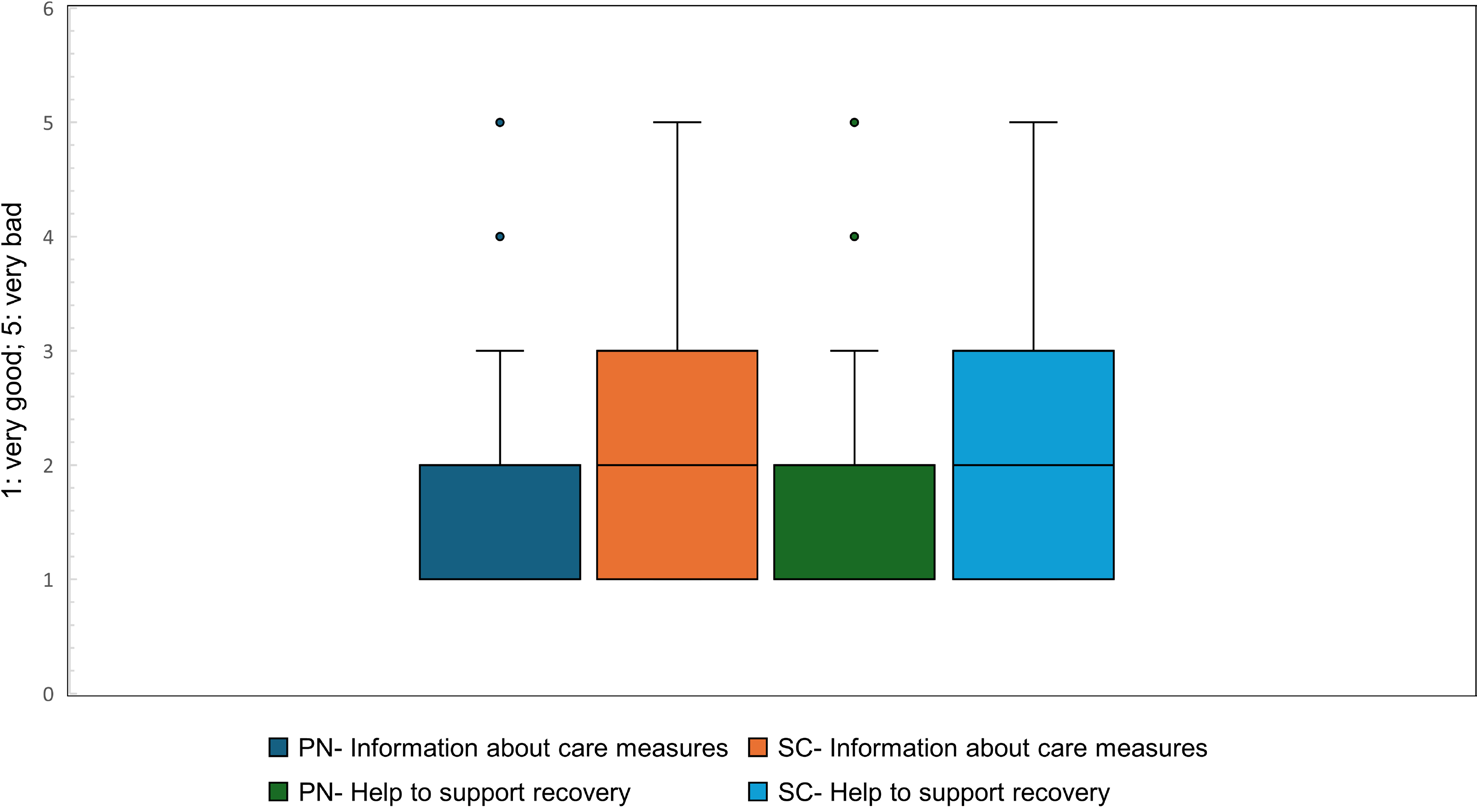
Boxplot of families' views on information about care measures and help to support recovery in PN and SC. Abbreviations: PN, primary nursing; SC, standard care.

Out of the 14 items concerning family satisfaction and nursing framework conditions, statistically significant differences were found in three (Table 1 and Figure 2). Family members of patients on PN ranked on a five‐point Likert scale (1: very good; 5: very bad) that they had received information about care measures in median with 1 (IQR: 1 to 2), compared to 2 (IQR: 1 to 3) in SC (*p* = 0.047). Moreover, they ranked their information to help support the patient's recovery on the same five‐point Likert scale differently in median between PN (1; IQR: 1 to 2) and SC (2; IQR: 1 to 3) (*p* = 0.01). Families stated that there was a permanent nurse as contact available on a three‐point Likert scale (1: yes, 2: partly, 3: no) in median with 2 (1 to 2) in PN and 2 (1 to 3) in SC (*p* = 0.004). Further results were similar between both groups (Table 2).

**TABLE 1:** Satisfaction of families and nursing framework conditions by study group.

	Overall^a^ *n* = 213	Primary nursing^a^ *n* = 63	Standard care^a^ *n* = 150	*p* ^b^ (*r*)	Unanswerable (*n*) (primary nursing/standard care)
Nurses responded to questions and wishes^c^	1 (1 to 1)	1 (1 to 1)	1 (1 to 1)	0.39	0/4
Impression of the professional skills of nurses^d^	1 (1 to 1)	1 (1 to 1)	1 (1 to 1)	0.58	3/5
Information from the nurses about daily routine^d^	2 (1 to 2)	2 (1 to 2)	2 (1 to 2)	0.85	6/30
Received information material about ICU stay^e^	3 (1 to 3)	2 (1 to 3)	3 (1 to 3)	0.71	5/20
Information about care measures^d^	1 (1 to 2)	1 (1 to 2)	2 (1 to 3)	0.047 (0.15)	10/35
Respect for patient's privacy^c^	1 (1 to 1)	1 (1 to 1)	1 (1 to 1)	0.54	3/8
Help to support recovery^d^	2 (1 to 3)	1 (1 to 2)	2 (1 to 3)	0.01 (0.2)	22/61
Discussion of worries and fears with the nurses^c^	1 (1 to 1)	1 (1 to 1)	1 (1 to 1)	0.87	1/13
Influence of families on patient's care^c^	1 (1 to 2)	1 (1 to 2)	1 (1 to 2)	0.57	21/65
Permanent nurse as contact available^e^	2 (1 to 3)	2 (1 to 2)	2 (1 to 3)	0.004 (0.5)	5/20
Felt taken seriously by nurses^c^	1 (1 to 1)	1 (1 to 1)	1 (1 to 1)	0.92	1/2
Explanation of how to use the room bell^e^	1 (1 to 2)	1 (1 to 1)	1 (1 to 2)	0.49	5/15
Availability of nurses via the room bell^c^	1 (1 to 1)	1 (1 to 1)	1 (1 to 1.5)	0.90	13/15
Evaluation of nursing care in the intensive care unit^d^	1 (1 to 1)	1 (1 to 1)	1 (1 to 1)	0.99	0/1

Abbreviation: ICU, intensive care unit.

^a^ Median with interquartile range.

^b^ Calculated by Wilcoxon rank test.

^c^ Scale 1–5, 1: yes, completely; 5: no, not at all.

^d^ Scale 1–5, 1: very good; 5: very bad.

^e^ Scale 1–3, 1: yes, 2: partly, 3: no.

**TABLE 2:** Support of physicians and interprofessional collaboration by study group.

	Overall^a^ *n* = 213	Primary nursing^a^ *n* = 63	Standard care^a^ *n* = 150	*p* ^b^ (*r*)	Unanswerable (*n*) (primary nursing/standard care)
Physicians responded to questions and wishes^c^	1 (1 to 1)	1 (1 to 2)	1 (1 to 1)	0.12	2/5
Spontaneous availability of physicians^c^	1 (1 to 2)	1 (1 to 2)	1 (1 to 2)	0.17	4/15
Discussion of worries and fears with physicians^c^	1 (1 to 2)	1 (1 to 2)	1 (1 to 1)	0.022 (0.1)	4/14
Collaboration of interprofessional staff (nurses, physicians, physiotherapists, psychologists, pastoral caregivers, …)^c^	1 (1 to 2)	1 (1 to 2)	1 (1 to 2)	0.98	15/28

^a^ Median with interquartile range.

^b^ Calculated by Wilcoxon rank test.

^c^ Scale 1–5, 1: yes, completely; 5: no, not at all.

### 4.2 Support of Physicians and Interprofessional Collaboration

All four items in the context of the support of physicians and the interprofessional collaboration were answered using a five‐point Likert scale (1: yes, completely; 5: no, not at all). Physicians' responses to questions and wishes were similar in both groups, as well as their spontaneous availability and the collaboration of the interprofessional staff (Table 2). There was a statistically significant difference in the discussion of worries and fears in both groups (median; IQR) (PN: 1; 1 to 2; SC: 1; 1 to 1; *p* = 0.022).

### 4.3 Environmental Factors

Family members ranked the reception of the hospital in median as very good and stated a complete recommendation of the hospital to friends or their relatives (Table S4). The PN and SC groups differed statistically significantly in their evaluation of the ICU room setup (median; IQR: 2; 1 to 2 vs. 1; 1 to 2; *p* = < 0.001).

Additional free‐text comments showed a heterogeneous, but mostly positive feedback and further wishes/recommendations of family members in PN (*n* = 24; Table S5) and SC (*n* = 50; Table S6).

## 5 Discussion

This study evaluated satisfaction of families and nursing framework conditions, support of physicians, interprofessional collaboration and environmental factors in PN, compared with individual nursing as SC on two ICUs using a validated questionnaire. Overall, there are positive results in both groups with isolated statistically significant differences in families' satisfaction in favour of PN.

To the best of our knowledge, we could not identify further studies with similar aims to those of our study. The study groups were not comparable, with around one third of patients' families having contact with PN. By comparison, during the 12‐month implementation period, roughly 150 patients received PN in the two ICUs [22]. This circumstance can be explained by the fact that the study began before the implementation of PN and, in particular, before the gradual transfer of patients to primary nurses during the implementation process [22].

In both groups, more than half of the participants were spouses. This means that mainly family members were visiting who were also likely to spend a lot of time with the patients outside the hospital. In both groups, family members reported visiting two or more times per week. Open visiting policies may further enhance their flexibility in this regard. Open visiting policies are a core recommendation in the FCC guideline [1] and also mentioned, for example, in the context of delirium prevention [31] and prevention of families' posttraumatic stress disorder [32]. Nevertheless, opening visiting policies are not currently in place on the participating ICUs, but it is an important step forwards to a better FCC [1, 12, 33]. A further research project is now planned in our hospital to assess barriers and facilitators to implement opening up visiting policies on both ICUs. Patients in PN and SC were not comparable. For example, patients in PN stayed in median more than twice the time on ICU and received around 350 h of mechanical ventilation, compared to around 50 h in SC. This shows that patients with more severe diseases are treated on ICU in the PN‐group. A longer stay on ICU, combined with the patient's critical illness, may also affect the family and their satisfaction with the primary nurse.

Overall results of families' satisfaction with PN are similar to SC. More information about care measures and a better help to support recovery by families are in line with the primary aims of PN [16] and available study results and conclusions of PN on ICU [18, 24, 34, 35]. Krüger et al. [26] reported comparable results in both items in a randomised feasibility trial (PN: *n* = 46, SC: *n* = 48). In our study, we were able to recruit a few more family members in PN and many more in SC. On the other hand, Krüger et al. [26] identified a statistically significantly better influence of families on patient care in PN, compared to SC. It is possible that the imbalance of participants in both groups led us to a different result. The patients of families in the PN group had a primary nurse in both ICUs. However, PN was not shown as fully implemented in the previous evaluation on ICU 2 [22]. Maybe this circumstance influenced our results, too. Moreover, Naef et al. [35] also reported challenges, like differences between units after implementation of PN, which have to be addressed by nursing care managers. Including family members in nursing visits could be another way to involve them more closely in the care process [36].

Nurses' professional skills and discussion of worries and fears were ranked very positively in PN and SC, while information by the nurses about the daily routine was positive in general. Primary nurses still use an additional flyer to inform patients and their families about their role in PN [22]. Brochures or booklets are also recommended in the context of bereavement [1, 37], delirium [38, 39] and general family support [37] on ICU. For example, communication, family presence and participation, as well as patient and family support are top‐ranked interventions in FCC by former patients, family members and healthcare professionals [40]. Moreover, communication is obligatory for interprofessional care on ICU [1, 8, 41, 42]. PN as a nursing organisation model combines these elements [3, 16]. An important key to obtaining information about the patient and related family is the structured assessment of nursing needs and social history [29, 32]. This approach also permits early identification of missing family background information, enabling the interprofessional team, including psychologists and pastoral counsellors, to offer timely support [43]. Further educational programmes are important and probably helpful for, especially, primary nurses to support nursing competencies of FCC [44, 45].

In both groups, the support of physicians and collaboration with interprofessional staff were rated positively. Interestingly, in SC, family members ranked discussion of worries and fears with the physicians more positively. Maybe individual situations led to this classification. Further studies should address this aspect. Individual interviews or focus group discussions would be suitable for this purpose.

Environmental factors were also ranked positively in both study groups. In general, family members would completely recommend the hospital to friends or their relatives. Interestingly, patients' rooms were rated statistically significantly better by family members in the SC group compared with the PN group. This is surprising because in PN, the structured assessment of nursing needs and social history is practiced to generate patient‐centred care with a personal environment on ICU, if possible. It is possible that individual circumstances led to this assessment in this case as well.

### 5.1 Strengths and Limitations

A strength of our study is that it specifically addresses family members of patients who are receiving PN or SC nursing care on two ICUs.

Nevertheless, our study also has its limitations. The participation rate in SC is twice as high as in PN. This was due to our study design and its start before the implementation of PN on both ICUs. A pre–post evaluation of the families' questionnaires prior to and following the implementation of PN was not possible because the response rate was too low. In addition, there may have been a bias due to the fact that SC is also practiced in parallel on both ICUs—for example, if patients stay < 3 days and there is a lack of nurses in the role of primary nurses. Furthermore, this situation may also have led to patients' family members in SC increasingly receiving nursing care similar to that provided in PN, even though no primary nurse was assigned to them. Both may introduce confounding effects. Nevertheless, both ICUs have at least 23 beds, and the large number of nurses likely minimises this effect.

The used questionnaire was validated in a geriatric setting [30] and not on ICU. This can reduce the validity of our results. However, the questionnaire was used for family members on ICU before [26]. Moreover, the study team was not always available for recruiting during visiting times. Furthermore, patients had greater severity of disease in PN. Another key point is that there may have been an influence of socially desirable behaviour, as the patients were still primarily receiving nursing care in the ICU during the study.

### 5.2 Implications for Practice and Further Research

Due to the specific medical focus of included ICUs, the results of our study can only be applied to other ICUs to a limited extent. The number of participants included could be increased with a larger nursing research group. Moreover, opening visiting policies on ICU may also influence the study results. For this reason, families should be addressed again in a further study after the introduction of open visiting policies.

Further studies should also focus on certified FCC ICUs as well as diverse areas of medical specialisation. Furthermore, a mixed‐methods study design enables a deeper understanding of families' views on PN. In addition to the used questionnaire, individual interviews or focus group interviews could be helpful to generate a deeper understanding of families' views on PN.

## 6 Conclusions

Family members in our study had contact to PN or SC as a nursing organisation model on ICU. The overall positive results in both groups may indicate a practiced FCC. In addition, the results are consistent with the descriptions of PN and individual nursing as SC [3, 16]. It is possible that the effect of switching from SC to PN does not show statistically significant differences between the two groups.

## Funding

The study was financed by the own funds of the Heart and Diabetes Center NRW, Ruhr University Bochum.

## Ethics Statement

The ethics committee of the Medical Faculty of the Ruhr University Bochum (2022–987), based in East Westphalia, approved the study on 18 November 2022.

## Consent

Participants provided written informed consent before the study commenced. Clinical Trial Registration: This study is registered at the German Clinical Trials Register as DRKS00030966.

## Conflicts of Interest

The authors declare no conflicts of interest.

## Supporting information


**Table S1:** Completed strengthening the reporting of observational studies in epidemiology (STROBE) checklist.
**Table S2:** Baseline characteristics of participants by study group.
**Table S3:** Baseline characteristics of patients by study group.
**Table S4:** Environmental factors by study group.
**Table S5:** Free text answers in primary nursing (*n* = 24).
**Table S6:** Free text answers in standard care (*n* = 50).

## Data Availability

The data that supports the results and findings of this study are available in the Supporting Information of this article.
