# Supplementary material for: Families' View on Primary Nursing in Intensive Care Units ‐ A Cross‐Sectional Study
Source: Nurs Crit Care. 2026 Jul 14;31(4):e70575. doi: 10.1111/nicc.70575 (PMC13366150; doi:10.1111/nicc.70575)
Supplement: Supplementary file 1 — Table S1: Completed strengthening the reporting of observational studies in epidemiology (STROBE) checklist. Table S2: Baseline characteristics of participants by study group. Table S3: Baseline characteristics of patients by study group. Table S4: Environmental factors by study group. Table S5: Free text answers in primary nursing (n = 24). Table S6: Free text answers in standard care (n = 50). [file NICC-31-0-s001.docx]

# Electronic Supplement

**Title**

Families' Views on Primary Nursing - A Cross Sectional Study

**Authors**

Lars Krüger, Francesco Squiccimarro, Thomas Mannebach, Almut Pörner, Benjamin Sarx, Christian Siegling, Esther Mertins, Tobias Becker, René Schramm, Jan Gummert, Volker Rudolph, Laura-Carina Kurz, Christian Höke, Franziska Wefer, Gero Langer

**Content**

Table S1: Completed Strengthening the Reporting of Observational Studies in Epidemiology (STROBE) Checklist

Table S2: Baseline characteristics of participants by study group

Table S3: Baseline characteristics of patients by study group

Table S4: Environmental factors by study group

Table S5: Free text answers in primary nursing (n=24)

Table S6: Free text answers in standard care (n=50)

- 1. **Table S1: Completed Strengthening the Reporting of Observational Studies in Epidemiology (STROBE) Checklist**

|  | Page(s)  (submitted manuscript) | Recommendation |
| --- | --- | --- |
| **Title and abstract** | Title p.  1-2 | (*a*) Indicate the study’s design with a commonly used term in the title or the abstract |
|  |  | (*b*) Provide in the abstract an informative and balanced summary of what was done and what was found |
| Introduction | | |
| Background/rationale | 3-4 | Explain the scientific background and rationale for the investigation being reported |
| Objectives | 4 | State specific objectives, including any prespecified hypotheses |
| Methods | | |
| Study design | 4 | Present key elements of study design early in the paper |
| Setting | 5 | Describe the setting, locations, and relevant dates, including periods of recruitment, exposure, follow-up, and data collection |
| Participants | 5 | (*a*) Give the eligibility criteria, and the sources and methods of selection of participants |
| Variables | 5-6 | Clearly define all outcomes, exposures, predictors, potential confounders, and effect modifiers. Give diagnostic criteria, if applicable |
| Data sources/ measurement | 6-7 | For each variable of interest, give sources of data and details of methods of assessment (measurement). Describe comparability of assessment methods if there is more than one group |
| Bias | 12 | Describe any efforts to address potential sources of bias |
| Study size | 5 | Explain how the study size was arrived at |
| Quantitative variables | 5-8 | Explain how quantitative variables were handled in the analyses. If applicable, describe which groupings were chosen and why |
| Statistical methods | 7-8 | (*a*) Describe all statistical methods, including those used to control for confounding |
|  |  | (*b*) Describe any methods used to examine subgroups and interactions |
|  |  | (*c*) Explain how missing data were addressed |
|  |  | (*d*) If applicable, describe analytical methods taking account of sampling strategy |
|  |  | (*e*) Describe any sensitivity analyses |
| Results | | |
| Participants | 8 | (a) Report numbers of individuals at each stage of study—eg numbers potentially eligible, examined for eligibility, confirmed eligible, included in the study, completing follow-up, and analysed |
|  |  | (b) Give reasons for non-participation at each stage |
|  |  | (c) Consider use of a flow diagram |
| Descriptive data | 8-9 | (a) Give characteristics of study participants (eg demographic, clinical, social) and information on exposures and potential confounders |
|  |  | (b) Indicate number of participants with missing data for each variable of interest |
| Outcome data | 8-9 | Report numbers of outcome events or summary measures |
| Main results | 8-9 | (*a*) Give unadjusted estimates and, if applicable, confounder-adjusted estimates and their precision (eg, 95% confidence interval). Make clear which confounders were adjusted for and why they were included |
|  |  | (*b*) Report category boundaries when continuous variables were categorized |
|  |  | (*c*) If relevant, consider translating estimates of relative risk into absolute risk for a meaningful time period |
| Other analyses | 8-9 | Report other analyses done—eg analyses of subgroups and interactions, and sensitivity analyses |
| Discussion | | |
| Key results | 9-10 | Summarise key results with reference to study objectives |
| Limitations | 12 | Discuss limitations of the study, taking into account sources of potential bias or imprecision. Discuss both direction and magnitude of any potential bias |
| Interpretation | 9-11 | Give a cautious overall interpretation of results considering objectives, limitations, multiplicity of analyses, results from similar studies, and other relevant evidence |
| Generalisability | 12-13 | Discuss the generalisability (external validity) of the study results |
| Other information | | |
| Funding | Title page | Give the source of funding and the role of the funders for the present study and, if applicable, for the original study on which the present article is based |

**Note:** An Explanation and Elaboration article discusses each checklist item and gives methodological background and published examples of transparent reporting. The STROBE checklist is best used in conjunction with this article (freely available on the Web sites of PLoS Medicine at http://www.plosmedicine.org/, Annals of Internal Medicine at http://www.annals.org/, and Epidemiology at http://www.epidem.com/). Information on the STROBE Initiative is available at www.strobe-statement.org.

- 1. **Table S2: Baseline characteristics of participants by study group**

|  | Overall  n=213 | Primary Nursing  n=63 | Standard Care  n=150 | Unanswerable (n)  (primary nursing/ standard care) |
| --- | --- | --- | --- | --- |
| Status of family member [n (%)]  spouse  daughter/son  other relative  friend  other  Frequency of ICU visits^1,a^  Current location of patient^1,b^ | 119 (55.87)  74 (34.74)  7 (3.29)  2 (0.94)  11 (5.16)  1 (1 to 1)  1 (1 to 1) | 40 (63.49)  16 (25.40)  2 (3.17)  0  5 (7.94)  1 (1 to 1)  1 (1 to 1) | 79 (52.67)  58 (38.67)  5 (3.33)  2 (1.33)  6 (4.00)  1 (1 to 1)  1 (1 to 1) | 0/0  0/0  0/0 |

^1^ Median with interquartile range

^a^ scale 1-4, 1 ≥ 2x/week; 2 once a week; 3 once a month; 4 <once a month

^b^ scale 1-2, 1 still on ICU; 2 patient has recently left ICU

Abbreviation: ICU, intensive care unit

- 1. **Table S3: Baseline characteristics of patients by study group**

|  | Primary Nursing  (n=63) | Standard Care  (n=150) |
| --- | --- | --- |
| Age (years)^1^  Female sex^2^  Hemodialysis^2^  ECMO use^2^  Mechanical ventilation (hours)^1^  Days on ICU^1^ | 64 (56 to 73)  23 (36.51)  39 (61.90)  9 (14.29)  353.65 (38.48 to 612.35)  26 (15 to 42) | 70 (63 to 78)  39 (26.0)  56 (37.33)  8 (5.33)  52.78 (14.77 to 371.85)  12 (7 to 24) |

^1^Median with interquartile range; ^2^n with % of patients

Abbreviations: ECMO, extracorporeal circulatory membrane oxygenation; ICU, intensive care unit

- 1. **Table S4:** **Environmental factors by study group**

|  | Overall^1^  n=213 | Primary Nursing^1^  n=63 | Standard Care^1^  n=150 | P-value^2^  (r) | Unanswerable (n)  (primary nursing/ standard care) |
| --- | --- | --- | --- | --- | --- |
| Evaluation of reception at hospital^a^  Evaluation of patient room setup on ICU^a^  Recommendation of hospital to friends or relatives^b^ | 1 (1 to 1)  1 (1 to 2)  1 (1 to 1) | 1 (1 to 1.25)  2 (1 to 2)  1 (1 to 1) | 1 (1 to 1)  1 (1 to 2)  1 (1 to 1) | 0.42  <0.001 (0.5)  0.71 | 7/12  5/7  0/6 |

^1^Median with interquartile range

^2^Calculated by Wilcoxon rank test

^a^scale 1-5, 1: very good; 5: very bad;

^b^scale 1-5, 1: yes, completely; 5: no, not at all

Abbreviation: ICU, intensive care unit

- 1. **Table S5: Free text answers in primary nursing (n=24)**

| Keep going as you have been; you are on a great path with the primary nursing concept. |
| --- |
| Conversations with the doctors are difficult because of the language barrier. I prefer speaking with the nurses because discussions with the doctors make me feel insecure and frightened. The doctors are doing a good job, and that is what matters most for the patient. |
| Extremely friendly and courteous staff. |
| Especially positive: the warmth, naturalness, empathy, sense of duty and professionalism of the nursing staff and the physicians. The primary nurse: excellent! |
| Especially positive: The availability via phone and the information provided on site. Very empathetic. |
| That it is sometimes very difficult to reach someone by phone when you cannot be there in person! |
| The team is top! |
| The vast majority of the primary nurses and doctors are very empathetic. That is not something to be taken for granted. I feel well cared for. |
| Accessibility is very good and the staff always polite and appreciative. In general, I would prefer visitors to wear masks and gowns, but that is not within the responsibility of the nursing staff. |
| Compassionate staff, always friendly. |
| It would be nice to receive more detailed information about the progress and the current condition — more plain, straightforward communication. It would also be desirable if everyone interacted with my husband in a more positive and empathetic way. |
| My mother initially felt very left alone with her worries and fears. She would have wished for more support and information from the beginning. |
| I am very satisfied with the nursing care. |
| I would strongly recommend having a conversation with the relatives immediately after the surgery. This should include information about the procedure itself and the resulting course of the illness. |
| Communication is very good. Fears are addressed. |
| My husband is still in a deep sleep, so I cannot say anything about the further care at this point. Up to now, everything has been fine. |
| The staff are very helpful and kind. |
| On a personal level, the tone is not always appropriate. To the world he may be just a man/patient, but to me he is the world. |
| Very good. The friendly and trusting manner toward patients and relatives. |
| As far as I can judge from the three hours I have spent sitting next to my husband’s bed, the nursing care is excellent. So far, there has been nothing that bothered me. I was particularly well and very empathetically informed by Dr. (XXX). I found both the telephone and in‑person conversations very reassuring. |
| A code word for requesting information by phone would be great. It would also be nice if some things could be discussed in a separate room rather than in passing. The nursing staff are very kind and helpful. |
| Super! |
| We are completely satisfied. Thank you to the doctors and the nursing staff of the intensive care unit. Thank you. |
| We know that our mother is in very good hands here, and what the nursing staff accomplishes is incredible. |

- 1. **Table S6: Free text answers in standard care (n=50)**

| All of the nurses and all of the doctors were/are very kind and competent! |
| --- |
| All very friendly and helpful. |
| Everything great. |
| Everything is good. It couldn’t be better. Thank you. |
| Everything was great! Thanks for everything. |
| Everything was really perfect! THANK YOU! |
| Everything was completely satisfactory! Thank you for everything!!! |
| A permanent presence by physicians would be helpful. The rest is fine. Tracheostomy information was provided, but then they didn’t report as promised (communication). |
| The physicians and nurses always helped ensure that our questions were answered and our fears were eased. She is in the best hands here, and it’s such a relief to know that. Even though they have so much to do, they remain so positive. |
| Explanation regarding the patient’s ability to perceive things (e.g. to what extent the patient can currently hear, see, etc.) |
| So far, I can’t say anything negative. |
| The reception staff are especially nice! |
| The professional staff are always friendly and consistently approachable. |
| The staff are very friendly and always helpful. One thing that could perhaps be improved is providing clearer information to family members during the first visit, so they don’t feel left on their own or worry unnecessarily. That’s not beneficial — not only for the relatives, but also for the patient. Even when they are asleep, patients still perceive what is happening around them. |
| The telephone at the entrance is used many times a day by different people, and the same goes for the door handles. I think it would be necessary to install a disinfectant dispenser next to the telephone. |
| The empathetic manner of the nursing staff in their interactions with patients and relatives is unique! |
| Communication with the physicians in the intensive care unit can improve. Diagnoses were presented completely without emotion, almost like news reports, and often left us feeling helpless and shocked. |
| I was very pleased with the nursing care my husband received. The physicians responded to my questions and wishes. Better mobile phone reception would be appreciated. |
| A coffee machine would be great. |
| I appreciated that everyone was very kind and really tried to listen when we had questions, even if they couldn’t be answered immediately. |
| The nurses always offer a smile, compassion, food, tea — everything you might need. It’s wonderful that you’re allowed to stay, even for longer, in an emergency situation. They really do everything possible to support you. |
| Overall, I am satisfied with the medical care. Nursing care: I would appreciate having one consistent contact person. Physicians: better coordination among the physicians and clearer transparency regarding the treatment plan. |
| Good: personal communication with the patient and clear announcements of all procedures. Bad: sometimes inconsistent interaction and engagement among different staff members. |
| I have the impression that every member of the nursing staff is doing their very best. |
| I have rarely experienced nursing staff taking so much time to answer questions or update me on the latest developments. Fantastic! |
| I have nothing to complain about, and I am glad and grateful that my husband is being so well cared for here. Thank you. |
| The individuality of the care provided was very noticeable in a positive way. The nursing staff also showed a very high level of empathy. |
| Every nurse should ask relatives about their concerns and questions. Every nurse demonstrated competence and expertise. Unfortunately, visiting hours are limited to the period from 2 p.m. to 6 p.m. |
| No remarks — we/I feel comfortable here and well cared for. |
| Communication between the social services team, doctors, patients, and relatives was unfortunately inadequate; better coordination would have been appreciated. |
| Medically, in terms of nursing care, and on a human level: excellent! Great praise to the hospital! The preparation for the situation was especially good! |
| My relative was excellently cared for. |
| My husband is in very good hands here, and that gives me a sense of security and trust as well. |
| In addition to the professional expertise, there is an exceptionally high level of humanity. The staff respond quickly to the needs of both patients and relatives, show empathy and attentiveness, and even anticipate potential problems. We could not imagine better care and have never experienced anything like it before. A big ‘thank you’. |
| Excellent staff |
| The nursing staff are generally very friendly, happy to provide information, dedicated, and highly skilled. |
| Respond more quickly to the call bell. |
| Very nice and open nursing team. After arranging it in advance, I was kindly allowed onto the ward before official visiting hours. I did not encounter any physicians during my two‑day visit. |
| A very calm and relaxed atmosphere, so that relatives and patients do not feel any stress. All of the nursing staff who cared for our family member were always friendly, professionally competent, helpful, and willing to listen. |
| You’re doing an excellent job. Please keep it up. Thank you for everything. |
| We have never experienced such good and competent care before. Thank you for everything! |
| Both the nurses and the doctors are fully committed. You can see it and feel it. Thank you for that. |
| Thank you! |
| Thank you very much for the excellent care. As a relative, I was able to go home feeling reassured because I knew my partner was in very, very good hands. Thank you! |
| Suggestion: a short written overview of the procedures as a helpful guide. Particularly positive: attentive, friendly, and kind staff. (Lid for the commode chair). |
| We are very, very, very happy that my father is being so well cared for here, and that everyone is so incredibly kind and friendly. Thank you so much! |
| We are very satisfied and grateful for everything the team here is doing for our mother. |
| We would like to express our thanks for the highly professional and friendly care and support. |
| Pay more attention to the wishes of the relatives. Otherwise everything is great! |
| There are not enough parking spaces. |
